# Chimeric Antigen Receptor T Cell Therapy and Its Significance in Multiple Myeloma

**DOI:** 10.7759/cureus.15917

**Published:** 2021-06-25

**Authors:** Jaskamal Padda, Khizer Khalid, Ujala Zubair, Mounika M Peethala, Varsha Kakani, Lakshmi Goriparthi, Abdulelah H Almanie, Ayden Charlene Cooper, Gutteridge Jean-Charles

**Affiliations:** 1 Internal Medicine, JC Medical Center, Orlando, USA; 2 Internal Medicine, Avalon University School of Medicine, Willemstad, CUW; 3 Family Medicine, Dow University of Health Sciences, Karachi, PAK; 4 Internal Medicine, Rajeev Gandhi Institute of Medical Sciences, Kadapa, IND; 5 Internal Medicine, Kakatiya Medical College, Warangal, IND; 6 Internal Medicine, Advent Health & Orlando Health Hospital/JC Medical Center, Orlando, USA

**Keywords:** chimeric antigen receptor t-cell therapy, car t cells, multiple myeloma, cd38, bcma, syndecan-1 (cd138), cd19

## Abstract

Multiple myeloma (MM) has a five-year prevalence worldwide of 230,000 people and is known as the second most common hematological malignancy within the United States. Extensive research has been conducted to gain a wide range of treatment strategies, providing hope to these patients. Combination therapy using chemotherapy, monoclonal antibodies, and immunomodulatory drugs are the current management of choice. After the introduction of chimeric antigen receptor (CAR) T cell therapy, promising results have been evidenced. In this therapy, T cells are derived from the patient and modified in-vitro to induce receptors that later target specific antigens when they are injected into patients. CAR T cells use three mechanisms to kill tumor cells: cytolytic pathways, cytokine release, and Fas/FasL axis. In this review, we highlight the different tumor markers targeted for therapy against multiple myeloma (MM). Target antigens for CAR T cell therapy include B-cell maturation antigen (BCMA), signaling lymphocyte activation molecule F7 (SLAMF7), CD38, CD138, CD19, immunoglobulin kappa light chain, orphan G protein-coupled receptor class C group 5 member D (GPRC5D). With the benefit of improving survival and prognosis, this therapy does carry a risk of some adverse events such as cytokine release syndrome, encephalopathy, infections, hypogammaglobulinemia, and tumor lysis syndrome.

## Introduction and background

Cancer is one of the major causes of mortality in the world, which has various treatment options attributed to it. As neoplastic cells exhibit a wide range of heterogeneity and develop resistance, new treatment strategies continue to evolve in cancer care such as chimeric antigen receptor (CAR) T cell therapy [1]. Multiple myeloma (MM) accounts for 1% of all neoplasms and 18% of all hematological cancers in the United States. It is a plasma cell malignancy that is associated with bone marrow clonal proliferation [2]. MM is the second most common blood cell cancer within the United States, with a five-year worldwide prevalence estimated at 230,000. It primarily occurs in the geriatric population with a median age of diagnosis at 66-70 years [3-4]. The diagnostic criterion of MM consists of ≥ 10% cancerous plasma cell concentration in the bone marrow with end-organ damage. If end-organ damage is not present, the diagnosis is based on ≥ 60% cancerous plasma cells in the bone marrow or ≥ 2 focal lesions on imaging studies [2]. The main treatment for MM is chemotherapy with monoclonal antibodies, immune-modulatory drugs, and proteasome inhibitors. Medical treatment with chemotherapeutic drugs in high doses and stem cell transplant has given decent outcomes with a median survival of about seven to nine years, with some exceeding 10 years. Despite these available therapies, MM is considered to be an incurable disease with expected relapses and recurrences. Fortunately, CAR T cell therapy has emerged as a revolutionary method in treating recurrent cases of MM [2-3].

## Review

CAR T cell therapy

CAR T cell therapy is the T cell modification technique in which the T cells are taken from the patient, modified in vitro to express the receptors that target the tumor-specific antigen, and then injected back into the patient. Patients of any human leukocyte antigen (HLA) type can be treated with CAR T cell therapy as the identification of tumor-specific antigen occurs without the influence of a major histocompatibility complex (MHC) [5].

More than two decades ago, Gross G et al. first explained the theory of targeting cancer cells with modified T cells [6]. The first-generation CARs were developed based on this principle. Trials on the efficacy of first-generation CAR T cell therapy showed effectiveness against murine tumors and in vitro models but did not give promising results in human ovarian cancer treatment. First-generation CARs did not have co-stimulatory molecules, which made the T cells prone to energy after the initial activation, resulting in failure of persistence in vivo. Continued studies resulted in the second generation of CARs, which had better potential. The expansion of T cells was done for 56 days ex vivo with added co-stimulation to decrease the number of poorly differentiated cells and to maintain the proliferative capacity. This eliminated the problem with first-generation CARs and helped in the maintenance of the required source of effector progeny. Subsequently, more research has been carried out to improve the CAR T cell delivery system. Modifications have been made according to the tumor-specific antigen for various hematological malignancies and solid cancers [7].

Implementation of CAR T cell therapy

The process of generating the CAR T cells begins with collecting the leukocytes from the patient by the apheresis. Then the cells are cultured to induce proliferation. The T cells are constitutionally altered to express chimeric antigen receptors using lentiviruses, retroviruses, or transposons. This transduction leads to the expression of chimeric antigen receptors on the T lymphocytes. CAR-expressing T cells are allowed to proliferate further. In the meantime, patients are pre-conditioned. Pre-conditioning is the process where the patient’s leukocytes are depleted using chemotherapy. This preconditioning enhances the CAR T cell activity and increases the success rate of this treatment. Following the preconditioning step, the transduced T cells are introduced back into the patient [7-8].

Chimeric antigen receptor constructs

The primary pathology of cancer lies in the failure to restrict tumor growth. The basic target of CAR T cell therapy is to achieve specific immunity against the tumor. This is achieved by the modification of T cells with a structure consisting of four important elements: a stimulatory molecule, a transmembrane domain, co-stimulatory molecules, and a single-chain variable (scFv) fragment.

**Figure 1:**
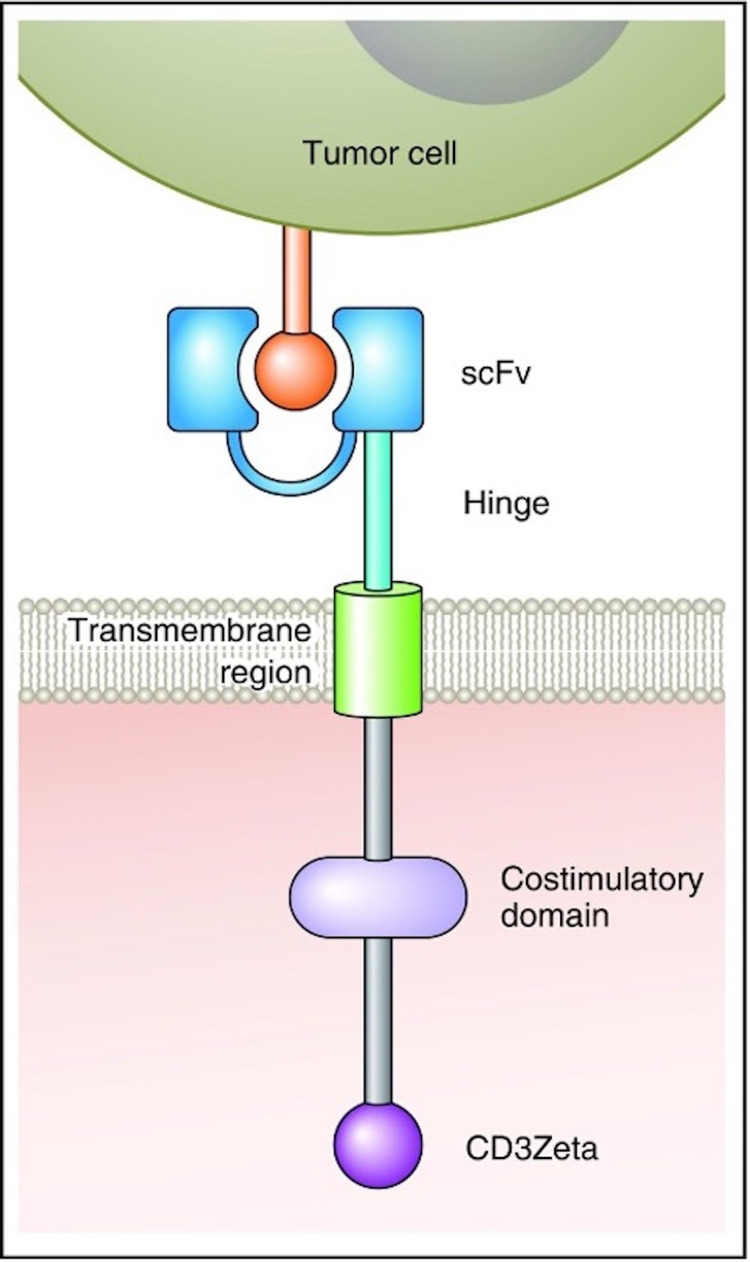
The CAR construct scFv - single-chain variable fragment [8] Permission to reproduce/reuse this figure was obtained from Elsevier Science & Technology Journals on June 08, 2021. License number: 1124410-1. CAR: chimeric antigen receptor

The scFv fragment, a fusion protein, is made from hybridoma cells. It is important in targeting the tumor cells and maintaining the effectiveness of CAR T cell therapy. The CAR is anchored to the T cell and fixed in place in the lipid bilayer of the cell with the help of the transmembrane domain, which also helps reinforce the bond between the target cell and the CAR T cell. Effective T cell activation is facilitated by stimulatory and co-stimulatory molecules. The T cell activation involves two steps in signaling. The first is the signal interaction between the antigen-presenting cell and T cell while the second is the co-stimulatory signal between the two cells [3]. Figure 1 explains the significant parts involved in the formation of CAR for effective functioning [8].

Killing of the tumor cells

Once the CAR T cells are injected into the patient, these cells mediate the killing of the tumor cells mainly by three mechanisms: cytolytic pathways, cytokine release, and Fas/FasL axis. The cytolytic pathways include the exocytosis of perforin and granzyme, which leads to lysis of the tumor cells and the expression of the membrane-bound TNF family ligands that induce apoptosis. The trimerization of the Fas receptor explains the Fas/FasL axis where the Fas ligand causes the activation of caspase 8. Caspase 8 is vital in the formation of caspase 3, a cell death mediator. While the direct tumor lysis due to T cell-tumor cell interaction is the primary mechanism of cell death in CAR T cell therapy, cytokine release from the activated CAR T cells further enhances anti-tumor capabilities. Cytokine release serves as a prominent mechanism of tumor cell killing mainly in solid tumors [9].

Target antigens and their importance

The goal of the treatment of any malignancy is to eliminate the cancerous tissue while preserving healthy tissues. The essential action of the chemotherapeutic agents is to eradicate cancer by interfering with the crucial actions of neoplastic cellular reproduction. Immunotherapeutic modalities of cancer treatment are highly selective to the tumor cells. Unfortunately, healthy cells of the human body still exhibit the same antigens that the tumor cells express, leading to some degree of autoimmune toxicity within normal tissues. The newly accepted CAR T cell therapy provides promising treatment to cure many advanced diseases, including MM. Previously conducted clinical trials in mice and humans have proven the benefits of CAR T cell therapy to localize and kill the malignant cells expressing specific, targeted antigens [10].

B-cell maturation antigen (BCMA)

Although MM is recognized to be an advanced hematological disease that is incurable in a variety of patients, CAR T cell therapy creates a new possibility of treatment for hematological malignancies. BCMA forms a therapeutic target in MM. It is a cell surface receptor expressed primarily in MM cell lines, particularly in plasma cells. BCMA intensity of expression is of high variability among patients diagnosed with MM. This is due to the shedding of BCMA from the plasma cell surface and its cleavage into a soluble form, making it detectable in circulation. Regarding BCMA’s role in MM, BCMA ligation assists in MM cell proliferation and survival. Accordingly, the promised proposal of therapies involving BCMA signaling blockage provides rationality in acclaiming it for target therapy in MM [11]. A previously conducted clinical trial by Cohen AD et al. concluded that BCMA-specific CAR containing D3ζ and 4-1BB signaling domain (CART-BCMA) infusions are clinically active with or without lymphodepleting chemotherapy in pretreated patients with MM [11]. This study project has reported the potentiality of CAR T cell therapy in MM. Twelve out of the 25 subjects achieved partial response while three subjects underwent a remission period of 11 months post-CART-BCMA therapy. Although elevated BCMA levels detected in the circulation are indicative of MM, the question remains whether there is an ultimate threshold for BCMA expression on the MM cell line required for the recognition and targeting of malignant cells [11]. CAR T cell therapy forms a different method of treating MM such as eliminating high levels of malignant plasma cells in the circulation that are potentially resistant to standard treatment. During the study observation, it is notable that some of the BCMA therapy candidates might need immunoglobulin replacement [12]. A study conducted by Ma T et al. demonstrated preclinical studies that have successfully proven the efficacy of BCMA-CAR T cells in murine xenograft models, in which the mouse had circulating MM cells. The MM cells were eradicated with the targeted therapy of anti-BCMA CAR T cells. The mouse’s life span was significantly prolonged after the trial [13]. Subsequently, multiple clinical trials are being conducted to prove the efficient utilization of anti-BCMA T cell therapy in MM. In progress to that, human clinical trials were conducted to estimate and prove the successful efficacy of anti-BCMA T cell therapy. In 2016, the first trial of investigation was done by Carpenter RO et al. for anti-BCMA-CAR second generation with the CD3/CD28 signal domain. The MM patients involved in the clinical trial had a uniform expression of BCMA investigated by flow cytometry or immunohistochemistry before initiation of the trial. This trial provided proof for the novel approach to MM therapy, further setting the benchmark for future clinical trials of investigation [13-14].

SLAMF7 and CD38

SLAMF7, which is also known as CD319/CS1/CRACC, is defined as a natural killer cell receptor that is involved in immune functions. It is normally expressed on hematologic cells such as plasma cells, B-cells, activated monocytes, and dendritic cells [8]. SLAMF7 is actively expressed in patients with MM. It plays a crucial role in the functions of the NK cell. Targeting SLAMF7 has shown benefits in patients with MM because it functions through many pathways to enhance the growth of multiple myeloma cells. Elotuzumab is a humanized anti-SLAMF7 antibody that has shown promising results among patients with MM. Elotuzumab acts by activating antibody-dependent cell-mediated cytotoxicity against MM cells by binding to the Fc region of antibodies by CD16 [15]. It has shown excellent results when used in combination with chemotherapy. When SLAMF7 is targeted along with CD38, the results are further enhanced. CD38 is actively expressed on myeloid, lymphoid, and plasma cells. CD38 has multiple mechanisms in the development of MM such as apoptotic functions, Fc-dependent immune effector functions, and immunomodulatory actions. CD38 functions as an ectoenzyme that is involved in NAD and NADP catabolism. Monoclonal antibodies against CD38 can diminish the myeloid-derived immunosuppressive cells along with B and T cells causing anti-tumor effects, which can be beneficial for patients with MM [16-17]. Daratumumab is a monoclonal antibody against CD38. Researchers have proved that it can induce antibody-dependent cell-mediated cytotoxicity, cell-dependent cytotoxicity, and programmed cell death. It has been shown to induce programmed cell death by the mechanism of cross-linking [18]. Isatuximab also acts against CD38. It is a humanized monoclonal antibody that has pro-apoptotic activities against MM cells. Radiotherapy against CD38 has also shown excellent results in multiple myeloma patients [19]. Therefore, the use of these immune-modulatory agents has improved the survival of multiple myeloma patients.

CD138

CD138 (syndecan-1) belongs to the syndecan family, which is required for cell-matrix and cell-cell interactions. It is a surface marker of malignant plasma cells and normal plasma cells in their early stages of differentiation. This antigen is expressed on endothelial, epithelial, and vascular smooth muscle cells. CD138 is associated with carcinogenesis in different stages such as metastasis, tumor invasion, angiogenesis, and cell proliferation. This allows for CD138 to be a viable target for CAR T cell therapy [20-21]. CD138 is more expressed in relapsed and refractory multiple myeloma (RRMM) rather than new cases, making CD138 an important target for anti-MM therapy [22]. A negative effect of CD138 CAR T cell therapy is the shedding of CD138 from the malignant plasma cell surface, making therapy ineffective. This proves the significance of treating with a combination of CAR target antigens in the treatment of RRMM [23]. CD138 helps in the adhesion of cells within the bone marrow, along with cell proliferation and survival. As the CD138 expression decreases, there is an increase in motility of cells leading to the dissemination of malignant plasma cells into circulation. Here, they are exposed to blood serum factors that aid in restoring the CD138 expression and assist in forming new foci of the tumor. Anti-CD138 therapy can decrease the tumor burden but increases the dissemination making it unsuccessful as single therapy [24]. Further investigation is needed for CD138 use in combination therapy as an effective treatment modality.

CD19

The US Food and Drug Administration (FDA) has approved the treatment of diffuse large cell lymphoma and acute lymphoid leukemia with CD19 CAR T cell therapy. CD19 expression is usually seen in myeloma stem cells and is absent in mature plasma cells. Premature cells with CD19 expression are responsible for disease progression, drug resistance, relapse, and reduced survival in MM [25]. CD19 CAR T cells (CTL019) target both CD19 and stem-cell antigens that are co-expressed in myeloma propagating cells (MPC), resulting in a favorable outcome [26]. Garfall AL et al. finished a prospective study using CTL019 after high-dose melphalan and salvage autologous hematopoietic stem cell transplantation (HSCT) in RRMM. The study was done with 10 patients who were infused with (1 to 5) ×107cells/kg CD19 CAR T cells two weeks after melphalan and a second autologous HSCT. Six out of ten demonstrated very good partial response, two achieved partial response, and two did not respond leading to disease progression [27]. Dual antigen-targeting therapy with anti-CD19/anti-BCMA showed a 100% overall response rate [28].

Immunoglobulin kappa light chain

Either kappa or lambda light chains are found to be expressed in mature B cells and B cell malignancies. Targeting the kappa light chain with CAR T cells in MM reduces toxicities, such as B-cell aplasia, hypogammaglobulinemia, and impaired humoral immunity, as seen with CD19 CAR T cell therapy, which is able to target the whole B cell lineage [29]. A research study conducted in MM patients resulted in a 70% positive detection rate of monoclonal kappa light chain antigen on the MM cell surface. There was no monoclonal lambda light chain antigen detected on the surface of MM cells. This signifies the use of kappa light chain antigen as a target antigen in treating MM [30]. Ramos CA et al. designed a study targeting kappa-restricted MM cells with kappa light chain CAR (KCAR) T-cells (NCT00881920), which is ongoing in a phase 1 clinical trial, last updated March 2021. Seven MM patients were infused with KCAR T cells after receiving cyclophosphamide. Four out of seven reached stable disease lasting from two to 17 months [31]. Kappa light chain immunoglobulins are secreted into the bloodstream, decreasing their expression over the MM cells and limiting the action of KCAR T cell therapy [29].

Orphan G-protein-coupled receptor class C group 5 member D (GPRC5D)

GPRC5D is a potential target for CAR T cell therapy because its expression in MM is associated with a poor prognosis. GPRC5D is expressed in hair cells, lung tissue, and bone marrow [32]. Its expression is limited in normal tissues, decreasing the adverse effects of anti-GPRC5D CAR T cell therapy. GPRC5D is a seven-pass transmembrane protein that does not shed in the serum as other surface antigens do such as BCMA and kappa light chain [33]. GPRC5D expression is independent of BCMA expression making anti-GPRC5D CAR T cells an alternative therapy in case of relapse after anti-BCMA therapy [34]. Table 1 summarizes the aforementioned target antigens and their role in CAR T cell therapy [8,11,15-17,20-21,25,29,32,34].

**Table 1:** Function and response of target antigens in CAR T cell therapy BCMA: B-cell maturation antigen, SLAMF7: signaling lymphocyte activation molecule F7, GPRC5D: orphan G-protein coupled receptor class C group member

Target Antigens	Function	Response
BCMA	Cell surface receptor.	BCMA ligation assists in multiple myeloma cell proliferation and survival.
SLAMF7	NK cell receptor. Expressed on B-cells, activated monocytes, and dendritic cells.	Targeted therapy against SLAMF7 acts by activating antibody-dependent cell-mediated cytotoxicity against multiple myeloma cells by binding to the Fc region of antibodies by CD16.
CD38	Ecto-enzyme expressed on myeloid, lymphoid, and plasma cells.	Involved in apoptotic function, Fc-dependent immune effector functions, and immunomodulatory actions.
CD138	Surface marker of malignant plasma cells and normal plasma cells in their early stages of differentiation. Expressed actively in relapsed and refractory multiple myeloma cases rather than new cases.	Required for cell-matrix and cell-cell interactions. Helps in the adhesion of cells with bone marrow along with cell proliferation and survival.
CD19	Expressed on myeloma stem cells and absent in mature plasma stem cells.	Responsible for disease progression, drug resistance, relapse, and reduced survival in multiple myeloma.
Immunoglobulin kappa light chain	Expressed in mature B-cells and B-cell malignancies.	Reduces toxicity such as B-cell aplasia, hypogammaglobulinemia, and impaired humoral immunity.
GPRC5D	Expressed in multiple myeloma associated with poor prognosis. Actively expressed on hair cells, lung tissue, and bone marrow.	Used as an alternative therapy in case of relapse after anti-BCMA therapy.

Toxicity of CAR T cell therapy

Cytokine Release Syndrome (CRS)

The onset of CRS is within two days to two weeks after the infusion of CAR T cell therapy. The pathophysiology is due to the activation of cytokines and interleukins. The acute phase reactant, C-reactive protein, is produced by interleukin-6, which is used as a laboratory marker for the onset and severity of CRS [35]. CRS toxicity can lead to adverse effects in multiple organ systems [36]. The grade of severity is determined by the combination of clinical symptoms. The management of grade one and two CRS is with supportive care. The addition of tocilizumab can be considered in case of hypotension or hypoxia which are not responsive to supportive care. For grade three CRS, corticosteroids can be prescribed in addition to tocilizumab [36].

Neurologic Toxicities

The onset of CAR T cell-related encephalopathy syndrome occurs within a week after cell infusion. The pathophysiology is due to endothelial dysfunction, elevated cytokine release, and increased blood-brain barrier permeability. Symptoms include ataxia, seizures, expressive aphasia, weakness, and disorientation. The severity of encephalopathy in pediatric patients <12 years of age is graded by the Cornell assessment of pediatric delirium (CAPD) and immune effector cell-associated encephalopathy (ICE) in >12 years of age [36]. Neurologic toxicity associated with CAR T cell therapy is reversible with supportive care. If neurological symptoms overlap with CRS, tocilizumab can be prescribed [37].

On Target/Off-Tumor Recognition

Most CAR T cell therapy targets share features of normal cells like the expression and presentation of antigens. When CAR T cells start to affect normal cells, it is known as “on-target/off-tumor” toxicity. An example of this is CD-19 being common in both normal B cells and as CAR T cell targets. Therefore, normal B cells can also be affected due to the shared expression of antigens causing B-cell aplasia and hypogammaglobulinemia. Prompt recognition and treatment of on-target/off-tumor toxicity should be initiated. Management includes monitoring, administering intravenous (IV) immunoglobulins, and antibiotic prophylaxis to prevent infection [37-39].

Infections

CAR T cell therapy decreases the patient’s immune system leading to the development of infections. CAR T cell therapy should not be administered to these patients until the symptoms have fully resolved. Prophylactic antibiotics, antifungals, and antivirals should be given as per institutional guidelines of CAR T cell therapy [39].

Tumor Lysis Syndrome

Rapid destruction of cells following CAR T cell therapy results in hyperuricemia, electrolyte disturbances, and renal injury. Management for increased levels of uric acid includes IV fluids, allopurinol, and rasburicase [36].

## Conclusions

The second most common hematological malignancy is MM, and it constitutes one percent of malignancies within the United States. MM has been contemplated to be an incurable disease due to its history of recurrence and relapse. Many therapeutic modalities are available, but due to the development of resistance, new treatments such as CAR T cell therapy are being investigated for the management of MM. Although CAR T cell therapy has many adverse effects, including CRS, infections, and tumor lysis syndrome, it has demonstrated promising results, which can be revolutionary in the treatment of MM. CAR T cell therapy collects the patient’s leukocytes and modifies them in vitro with the help of an inactivated virus. This modification allows for chimeric antigen receptors to be produced, which are then infused back into the patient. Now, CAR T cells can locate the target antigens to eliminate these neoplastic cells. Research has been conducted on many antigens such as BCMA, SLAMF7, CD38, etc. Further investigation is needed to identify and manage the long-term complications of CAR T cell therapy, recognize other viable targets, along with investigating the outcomes of combination/dual-target antigen therapy. Consequently, allowing for increased success rates with this novel treatment modality.
